# Comparative Transcriptomic Analysis Reveals the Underlying Mechanism of *Piriformospora indica*-Enhanced Root Rot Resistance in *Gerbera hybrida*

**DOI:** 10.3390/plants15121771

**Published:** 2026-06-08

**Authors:** Dingquan Huang, Yan Yang, Huan Wu, Jie Pan, Yongyan Zhang, Chunzhen Cheng

**Affiliations:** 1 Key Laboratory of Genetics, Breeding and Multiple Utilization of Crops, Ministry of Education, Fuzhou 350002, China; 2 Key Laboratory of Biological Breeding for Fujian and Taiwan Crops, Ministry of Agriculture and Rural Affairs, Fuzhou 350002, China; 3 College of Agriculture, Fujian Agriculture and Forestry University, Fuzhou 350002, China; 4 College of Horticulture, Shanxi Agricultural University, Taiyuan 030031, China; 5 College of Horticulture, Fujian Agriculture and Forestry University, Fuzhou 350002, China

**Keywords:** comparative transcriptomics, *Gerbera hybrida*, *Piriformospora indica*, resistance mechanism, root rot disease

## Abstract

*Gerbera hybrida*, a model plant for studying the development and evolution of complex inflorescences, has been recognized as a valuable source of colorants and natural coatings. Previously, we demonstrated that inoculation with *Piriformospora indica*, an endophytic fungus, could enhance the resistance of gerbera to *Phytophthora cryptogea,* which causes root rot disease. Here, to dissect the underlying molecular mechanism, comparative transcriptomic analyses of gerbera roots from *P. indica*-colonized (PI), *P. cryptogea*-inoculated (PC), and *P. cryptogea*-inoculated *P. indica*-colonized (PP), along with the non-inoculated control (CK) groups were conducted. In total, we identified 15,763 differentially expressed genes (DEGs) among CK vs. PI, CK vs. PC, and PI vs. PP comparisons, together with PC vs. PP comparisons. Of them, 743 DEGs encode transcription factor (TF) proteins, with the *AP2/ERF* family members occupying the majority. An *ERF* gene (*GhERF*) was identified as one of the 30 common DEGs among the four comparisons. Notably, its transient overexpression in tobacco leaves reduced the lesion area caused by *P. cryptogea* inoculation to 65.36% of the empty vector control. Many defense response- and secondary metabolism-related DEGs were expressed higher in the PP group than in the PC group. Moreover, *P. indica*-colonization up-regulated the expression of hormone signaling- and MAPK signaling-related genes, thereby activating the defense responses against the pathogen infection. Our study is helpful for understanding the molecular mechanism underlying *P. indica*-enhanced root rot resistance in gerbera.

## 1. Introduction

*Piriformospora*/*Serendipita indica* is a cultivable endophytic fungus exhibiting arbuscular mycorrhizal fungi-like (AMF-like) growth-promoting effects [1,2]. It establishes a mutualistic symbiosis with a broad range of host plants, improves nutrient uptake, and induces systemic resistance against diverse phytopathogens [3,4]. Given its axenically cultivable and widespread host plus well-established genetic transformation system, it has been recognized and applied as a research model for plant–fungi interactions [5]. Besides its plant-growth-promoting effects [6], *P. indica* colonization in plant roots can also enhance soil urease and phosphatase activities, improve plant utilization efficiency of soil nutrients, and increase soil catalase activity to alleviate the toxicity of hydrogen peroxide [7].

Evidence has shown that *P. indica* can efficiently enhance plants’ resistance to a variety of abiotic and biotic stresses [8]. Over the past two decades, its capacity to improve resistance to a range of biotic stresses caused by fungi, bacteria, nematodes, and insects, among others, has drawn significant attention from scientists [9,10]. It should be noted that distinct mechanisms are involved when *P. indica* resists colonization by different pathogens in plants [11]. For example, *P. indica*-colonized barley exhibited obvious milder disease incidences in leaves after powdery mildew infection compared to the mock, and activated systemic acquired resistance [12]. *P. indica* exhibited suppressive effects on the pathogenicity of *Fusarium verticillioides*, thereby directly inhibiting the colonization and spreading of the pathogen [13]. Although *P. indica* colonization can enhance the resistance of chickpea to gray mold, it has no antagonistic effect on the gray mold pathogen *Botrytis cinerea* [14]. Likewise, *P. indica* has been reported to lack direct inhibitory activity against *F. oxysporum* f. sp. *cubense* tropical race 4 [15] and *Phytophthora cryptogea* [16]. Nevertheless, it can improve host resistance to the corresponding diseases by modulating plant defense responses. Moreover, some studies have even demonstrated that *P. indica* mycelial growth can be significantly inhibited by pathogens [11].

*Gerbera hybrida* is not only a model plant for studying the development and evolution of complex inflorescences [17,18], but also a valuable raw material for producing colorants and antioxidants [19,20]. Considering its enormous economic value, the cultivation area has expanded rapidly. However, modern gerbera cultivars generally exhibit poor disease resistance, making them particularly vulnerable as plantation scales expand. Among these diseases, root rot disease caused by soil-borne *P. cryptogea* or *F. oxysporumis* is considered to be the most devastating one [21]. The infected plants exhibit wilting, chlorosis, root necrosis, vascular discoloration, and early death, all of which impact plant quality and market value. Due to restrictions in the chemical control of these soil-borne pathogens and widespread infection emerging as a critical constraint to the healthy development of the gerbera industry, biological control methods are especially urgent [22]. In one of our previous studies, we demonstrated that although *P. indica* has no significant antagonistic effect on *P. cryptogea*, it can enhance the efficacy of gerbera to withstand root rot by enhancing activities of antioxidant enzymes, stimulating accumulations of growth-promoting phytohormones 3-indoleacetic acid (IAA) and gibberellic acid (GA), as well as by inhibiting biosynthesis of senescence/stress-associated phytohormones abscisic acid (ABA) and jasmonic acid (JA) [16]. However, the underlying molecular mechanism is still obscure. In our present study, comparative root transcriptomic analysis of *P. indica* non-colonized (CK) and colonized (PI) gerbera before and after *P. cryptogea* inoculation (PC and PP) was conducted. Moreover, the function of the *P. indica* and *P. cryptogea* inducible *ethylene response factor* (*ERF*) gene, *GhERF*, was verified by employing a tobacco leaf transient overexpression-based *P. cryptogea* resistance assay. Our study is helpful for understanding how *P. indica* enhances gerbera resistance against root rot disease and provides a basis for future application of this fungus in the gerbera industry.

## 2. Results

### 2.1. RNA-Seq Analysis and Identification of DEGs

**Figure 1:**
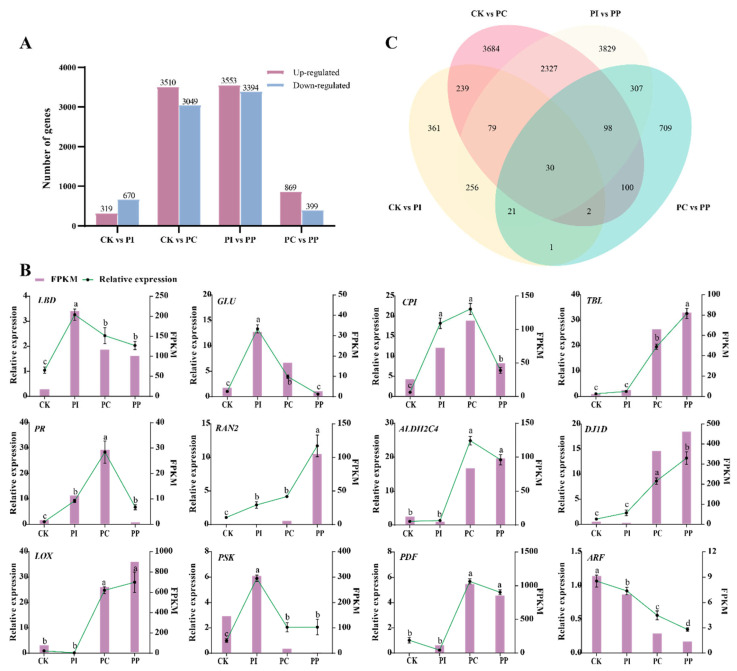
Statistical analysis and gene transcriptional validation results of DEGs from CK vs. PI, CK vs. PC, PI vs. PP, and PC vs. PP comparisons. (**A**) Numbers of DEGs in the four comparisons. (**B**) RT-qPCR analysis results of selected DEGs. Different letters above the columns indicate significant difference (*p* < 0.05). (**C**) Venn diagram for DEGs identified from the four comparisons. LBD: lateral organ boundaries domain protein; GLU: β-Glucosidase; CPI: cysteine protease inhibitor; TBL: trichome birefringence-like; PR: pathogenesis-related protein; RAN2: RAS-related GTP-binding nuclear protein 2; ALDH2C4: aldehyde dehydrogenase family 2 member C4; DJ1D: DJ-1 protein homolog D; LOX: lipoxygenase; PSK: phytosulfokine; PDF: plant defensin; and ARF: auxin response factor.

A total of 803.98 Mb clean reads were obtained from 12 cDNA libraries (CK1~3, PC1~3, PI1~3, and PP1~3). The base numbers in each library ranged from 6.34 Gb to 7.03 Gb, with Q20 and Q30 values greater than 97% and 89% (Table S1), respectively. In total, we identified 70,950 genes expressed in at least one library. Among them, 15,763 were identified as DEGs (Figure 1A), including 989 DEGs in CK vs. PI (319 up-regulated and 670 down-regulated) (Table S2), 6559 DEGs in CK vs. PC (3510 up-regulated and 3049 down-regulated) (Table S3), 6947 DEGs (3553 up-regulated and 3394 down-regulated) in PI vs. PP (Table S4), and 1268 DEGs (869 up-regulated and 399 down-regulated) in PC vs. PP comparisons (Table S5). Notably, 30 genes were identified as common DEGs among all four comparisons (Figure 1C), including genes encoding disease resistance proteins, POD1, ERF, and so on. Furthermore, most of these genes were identified as homologous genes of stress-responsive genes in Arabidopsis (Table 1).

Real-time quantitative PCR (RT-qPCR) analysis of 12 selected DEGs involved in plant growth and development, metabolites and phytohormone metabolism, and defense response showed that their transcriptional patterns were mostly consistent with our transcriptome data (Figure 1B), indicating that the transcriptome sequencing results were reliable.

### 2.2. Gene Ontology (GO) Enrichment Analysis Results of DEGs

**Figure 2:**
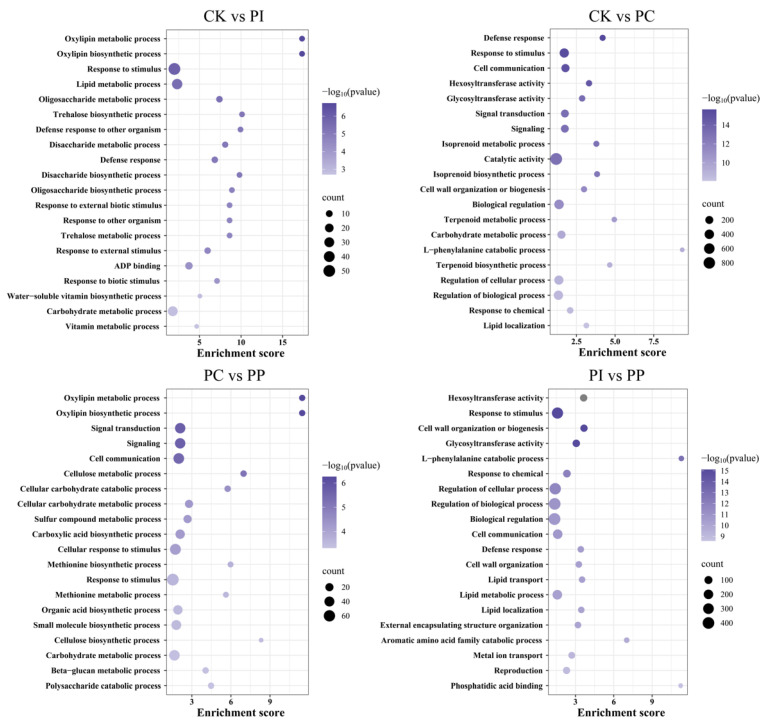
Top 20 significantly enriched GO terms of DEGs from the four comparisons.

GO enrichment analysis of all DEGs, up-regulated DEGs, and down-regulated DEGs from each comparison was performed (Figure 2; Tables S6–S8).

For the CK vs. PI comparison, DEGs were significantly enriched in 38 biological process (BP) terms (including ‘oxylipin metabolic process’, ‘oxylipin biosynthetic process’, ‘response to stimulus’, ‘lipid metabolic process’, and so on) and one molecular function (MF) term (‘ADP binding’) (Table S6); up-regulated DEGs were remarkably enriched in seven BP terms and three MF terms (Table S7); and down-regulated DEGs were significantly enriched in 32 BP terms and one MF term (‘ADP binding’) (Figure S1A; Table S8).

**Figure 3:**
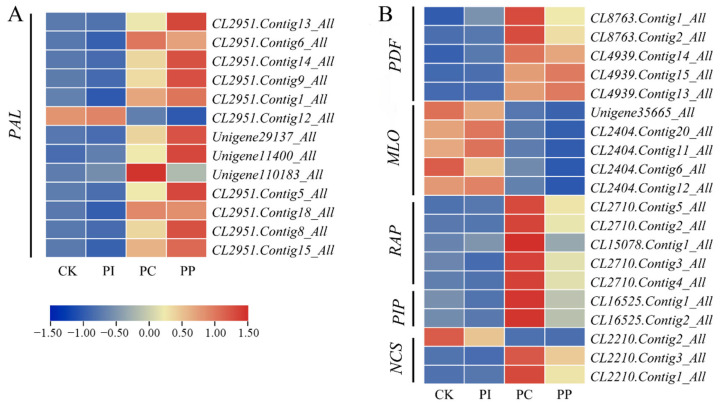
Gene expression heat map for the ‘L-phenylalanine catabolism’ (**A**) and ‘defense response’-related (**B**) DEGs. PAL: phenylalanine ammonia lyase; PDF: plant defensin; MLO: mildew resistance locus O; RAP: root allergen protein; PIP: PAMP-induced secreted peptide; and NCS: norcoclaurine synthase.

For the CK vs. PC comparison, DEGs were significantly enriched in 182 BP (including ‘response to stimulus’, ‘defense response’, ‘cell communication’, ‘L-phenylalanine catabolic process’, and so on), 18 MF, and 19 cellular component (CC) terms (Table S6); up-regulated DEGs were significantly enriched in 176 BP terms, 22 MF terms, and 21 CC terms (Table S7); and down-regulated DEGs were significantly enriched in 139 BP terms, 16 MF terms, and three CC terms (Table S8). It is noteworthy that the 13 DEGs involved in the ‘L-phenylalanine catabolic process’ term were all *phenylalanine ammonia lyase (PAL)* genes. Except for one *PAL* (CL2951.contig12_All), all the *PALs* were highly expressed in the roots of the PC and PP groups. The ‘defense response’-related DEGs included many broad-spectrum antimicrobial factor-associated genes, such as *plant defensin* (*PDF*), *PAMP-induced secretory peptide* (*PIP1*), *mildew resistance locus O* (*MLO11*), *root allergen protein* (*RAP*), and *norcoclaurine synthase* (*NCS*) (Figure 3). All these DEGs, excluding four *MLO* genes and one *NCS* gene, were highly expressed in the PC and PP groups, and most of them demonstrated the highest expression in the PC group.

For the PI vs. PP comparison, DEGs were significantly enriched in 194 BP (including ‘response to stimulus’, ‘L-phenylalanine catabolic process’, and so on), 11 MF, and three CC terms (Table S6); up-regulated DEGs were significantly enriched in 118 BP, 11 MF, and seven CC terms (Table S7); and down-regulated DEGs were significantly enriched in 19 BP terms and one MF term (Table S8).

For the PC vs. PP comparison, DEGs were significantly enriched in 34 BP terms (including ‘signaling’, ‘oxylipin metabolic process’, ‘oxylipin biosynthetic process’, and so on) (Table S6); up-regulated DEGs were significantly enriched in 21 BP terms (Table S7); and down-regulated DEGs were significantly enriched in 28 BP and two MF terms (Table S8).

Remarkably, DEGs of the four comparisons were all significantly enriched in ‘oxylipin biosynthetic process’, ‘oxylipin metabolic process’, ‘response to stimulus’, ‘carbohydrate metabolic process’, ‘carboxylic acid biosynthetic process’, ‘cell communication’, ‘regulation of cellular process’, ‘signal transduction’, and ‘signaling’ terms. DEGs of the CK vs. PC and PI vs. PP comparisons shared the most common enriched GO terms, including 72 BP terms, seven MF terms, and three CC terms, suggesting that the *P. cryptogea* infection greatly influenced the root transcriptome profiling of both *P. indica* colonized and non-colonized gerbera.

### 2.3. KEGG Enrichment Analysis Results of DEGs

**Figure 4:**
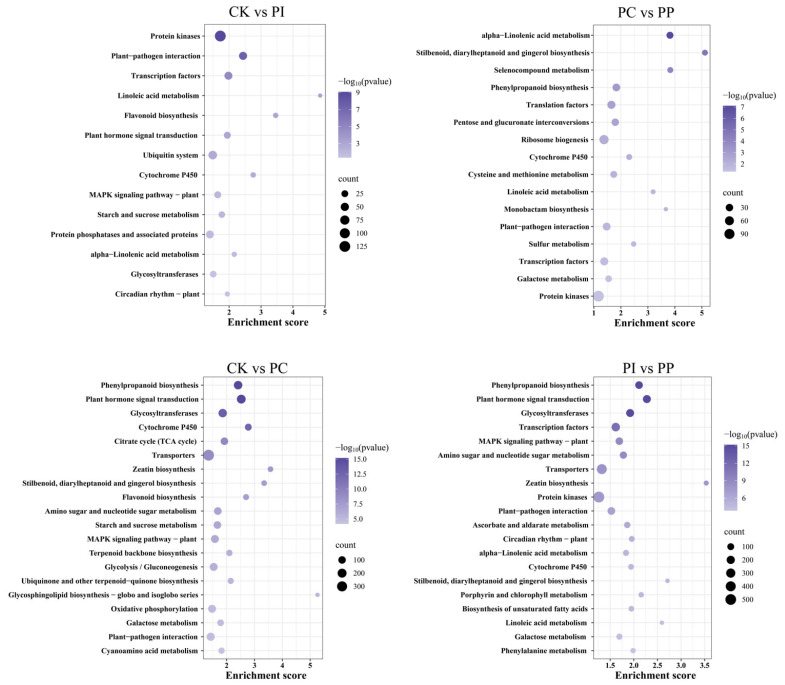
KEGG enrichment analysis results of DEGs from four comparisons. Only the top 20 enriched pathways were displayed for each comparison.

KEGG enrichment analysis of DEGs from each comparison was further analyzed (Figure 4). The DEGs and the up-regulated and down-regulated DEGs of the CK vs. PI comparison were significantly enriched in nine, four, and seven pathways (Figure S1B), respectively.

For the CK vs. PC comparison, DEGs were significantly enriched in 46 pathways (‘phenylpropanoid biosynthesis’, ‘plant hormone signal transduction’, ‘glycosyltransferases’, ‘MAPK signaling pathway-plant’, and so on) (Table S9); up-regulated and down-regulated DEGs were significantly enriched in 44 and 18 pathways (Table S10), respectively. The ‘phenylpropanoid biosynthesis’-associated DEGs primarily included genes encoding 4-coumarate-CoA ligase (4CL), caffeic acid oxygen methyltransferase (COMT), mannitol dehydrogenase (ELI), and telomere repeat-binding protein (TRP). DEGs involved in the ‘plant hormone signal transduction’ pathway included several auxin biosynthesis and signaling transduction-related genes, such as *ARF*, *IAA*, *SAUR,* and *TIFY* (Figure S1B). A variety of *TF* genes, such as *ERFs*, *MYCs*, and *bHLHs*, were identified as DEGs in this comparison.

For the PC vs. PP comparison, DEGs were significantly enriched in ‘alpha-linolenic acid metabolism’, ‘stilbenoid diarylheptanoid and gingerol biosynthesis’, ‘selenocompound metabolism’, ‘phenylpropanoid biosynthesis’, ‘translation factors’, and ‘pentose and glucuronate interconversions’ pathways (Table S9); up-regulated and down-regulated DEGs were significantly enriched in four and eight pathways (Table S10 and Table S11), respectively.

For the PI vs. PP comparison, DEGs were significantly enriched in 40 pathways (Table S9); up-regulated and down-regulated DEGs of the PI vs. PP comparison were significantly enriched in 11 and 18 pathways (Table S10 and Table S11), respectively.

Particularly, ‘cytochrome P450’, ‘linoleic acid metabolism’, ‘flavonoid biosynthesis’, ‘protein kinases’, ‘transcription factors’, ‘plant hormone signal transduction’, and ‘plant–pathogen interaction’ were commonly significantly enriched by DEGs of CK vs. PI, CK vs. PC, and PI vs. PP comparisons. The ‘protein kinases’ pathway was commonly significantly enriched by down-regulated DEGs from all four comparisons. The ‘linoleic acid metabolism’-associated DEGs of all four comparisons included 29 *LOX* genes (Figure S2). ‘Alpha-linolenic acid metabolism’, ‘phenylpropanoid biosynthesis’, and ‘stilbenoid, diarylheptanoid, and gingerol biosynthesis’ pathways were commonly significantly enriched by DEGs of CK vs. PC, PI vs. PP, and PC vs. PP comparisons. The ‘translation’ pathway was commonly significantly enriched by DEGs of CK vs. PI and CK vs. PC comparisons. The ‘ubiquitin system’ pathway was commonly significantly enriched by DEGs of CK vs. PI and PI vs. PP comparisons.

**Figure 5:**
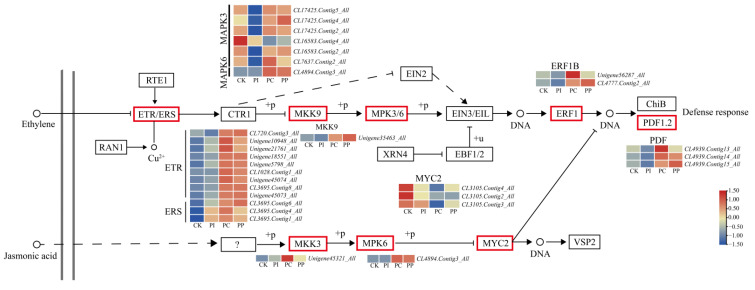
Diagram for the MAPK signaling pathway-related DEGs. Solid and dashed arrows represent direct and indirect reactions, respectively; red and black boxes indicate the presence and absence of DEGs encoding these proteins, respectively. ETR: ethylene receptor; ERS: ethylene response sensor; MKK: mitogen-activated protein kinase kinase; MPK: mitogen-activated protein kinase; ERF: ethylene-responsive transcription factor; and PDF: plant defensin.

Notably, DEGs of CK vs. PC and PI vs. PP comparisons shared the maximum amount of commonly significantly enriched pathways (18). The MAPK signaling pathway was significantly enriched in the CK vs. PC and PI vs. PP comparisons. It was revealed that the expression levels of MAPK signaling-related DEGs involved in the ethylene (ET) signal transduction pathway were prominently higher in the PC than in the PP group (Figure 5).

### 2.4. Analysis of Differentially Expressed TF Genes and Functional Study of GhERF

**Figure 6:**
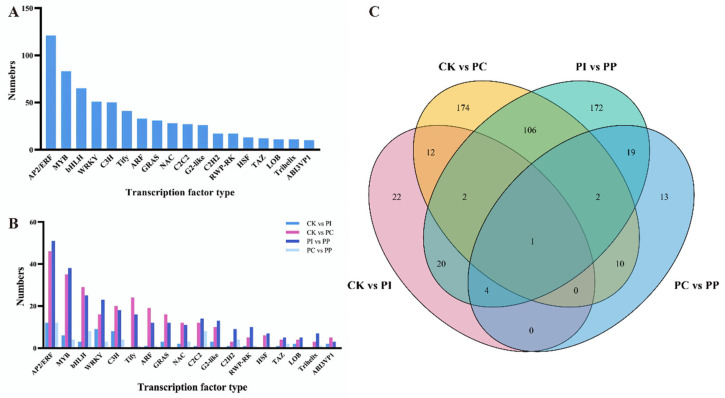
Statistics of DEGs encoding transcription factors (TFs). (**A**) The number of TFs encoding DEGs from the four comparisons; (**B**) the number of DEGs encoding TFs in each of the four comparisons; and (**C**) Venn diagram of differentially expressed *TF* genes in four comparisons. The *ERF* gene (CL12783.Contig2_All) was identified as the common differentially expressed *TF* gene among the four comparisons.

As DEGs from CK vs. PI, CK vs. PC, and PI vs. PP comparisons were all found to be enriched in the ‘transcription factors’ pathway, we further focused on the 743 TF-encoding DEGs (4.71% of the total DEGs), including 61 DE *TFs* in the CK vs. PI comparison, 307 in the CK vs. PC comparison, 326 in the PI vs. PP comparison, and 49 in the PC vs. PP comparison. These *TF*s belong to 51 gene families, among which *AP2/ERF* accounted for the majority (in total of 121), followed by *MYB* (83), *bHLH* (65), and *WRKY* (51) (Figure 6).

**Figure 7:**
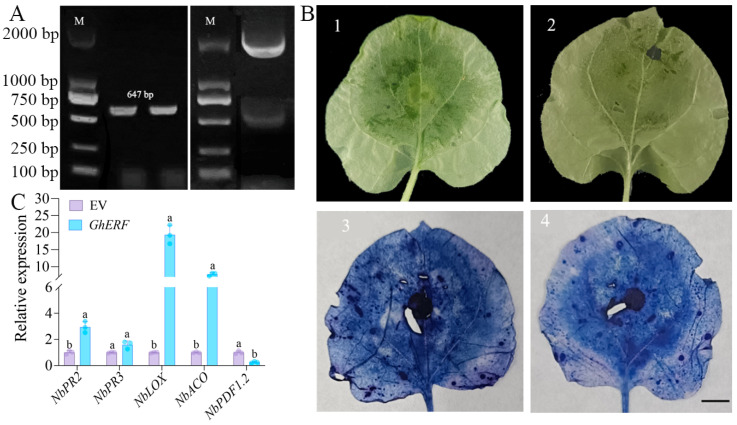
Functional analyses of *GhERF* employing tobacco leaf transient overexpression-based pathogen resistance assays. (**A**): Electrophoresis results for *GhERF* amplification products (left) and double-digested pCAMBIA1301-GhERF recombinant plasmids (right); M: marker. (**B**) Phenotype and trypan blue staining results of tobacco leaves at 3 days after *P. cryptogea* inoculation; 1: control; 2: tobacco leaf overexpressing *GhERF*; 3: control tobacco leaf after trypan blue staining; and 4: tobacco leaf overexpressing *GhERF* after trypan blue staining (Bar = 1 cm). (**C**) Transcriptional analysis results of defense-related genes in tobacco leaves after *P. cryptogea* inoculation. Different letters above columns indicate significant difference (*p* < 0.05). ERF: ethylene response factor; PR: pathogenesis-related protein; LOX: lipoxygenase; ACO: 1-aminocyclopropane-1-carboxylic acid oxidase; and PDF: plant defensin.

One *ERF* gene (CL12783.Contig2_All) was identified as one of the 30 overlapping DEGs in the four comparisons (Figure 6C). Its expression levels were significantly down-regulated in the PI and PC groups, while peaking in the PP group. This pattern suggests that the gene is specifically induced by the interplay between *P. indica* and *P. cryptogea*, rather than by either fungus alone. We successfully cloned the 647 bp cDNA sequence of *GhERF* (Figure 7A), with a 630 bp coding sequence (CDS) encoding a putative protein with 209 amino acids. To investigate its function in the gerbera resistance to *P. cryptogea* infection, we transiently overexpressed its CDS in *N. benthamiana* leaves (Figure 7B). Then, leaves overexpressing *GhERF* were inoculated with *P. cryptogea*. After 72 h of *P. cryptogea* inoculation, the mock leaves displayed obvious diseased symptoms. However, the symptoms in *GhERF*-overexpressing leaves were apparently milder, with the lesion area accounting for approximately 65.36% of the control (Figure 7B). The Trypan blue staining results also showed that overexpression of *GhERF* could inhibit the growth of *P. cryptogea*. To investigate the underlying mechanism, the transcriptional levels of defense-related genes (*NbPR2*, *NbPR3*, *NbACO*, *NbLOX*, and *NbPDF1.2*) in tobacco leaves were evaluated. The RT-qPCR results revealed that the expression of *NbPR2*, *NbACO*, and *NbLOX* in tobacco leaves overexpressing *GhERF* was significantly up-regulated, accounting for 2.94-, 7.78-, and 19.41-fold of EV, respectively. However, the expression level of *NbPDF1.2* in tobacco leaves overexpressing *GhERF* was only 25.82% of EV (Figure 7C).

## 3. Discussion

### 3.1. P. indica Colonization Up-Regulates the Expression of Defense Response- and Secondary Metabolism-Related Genes in Gerbera Roots

In this study, we compared the root transcriptome profiling of *P. indica*-colonized and non-colonized gerbera before (PI and CK) and after *P. cryptogea* inoculation (PP and PC), and identified 15,763 DEGs among the four groups. *P. cryptogea*-responsive DEGs were markedly greater in number than the *P. indica*-responsive genes, and 30 genes were identified as common DEGs among the four comparisons. Of the 30 common DEGs, there are several disease resistance-related genes, such as *disease resistance proteins*, *LOX3.1*, *POD,* and *CPK*. Of the ‘defense response’-related DEGs, several broad-spectrum antimicrobial factor-related genes were included, including *PDF*, *PIP1*, *MLOs*, *RAP,* and *NCS*. Among them, only *MLOs* were down-regulated in the PC and PP groups, and their expression levels in the PC group were all higher than in the PP group. It is well known that *MLOs* confer negative functions in plant disease resistance [42]; their down-regulation in PP roots and significantly lower expression levels in PP indicated that the colonization of *P. indica* enhanced the defense responses in gerbera root.

Secondary metabolites serve as potent regulators in plant growth and defense [43]. In this study, DEGs of the CK vs. PI, CK vs. PC, and PI vs. PP comparisons were significantly enriched in ‘flavonoid biosynthesis’, DEGs of the CK vs. PC, PI vs. PP, and PC vs. PP comparisons were simultaneously enriched in ‘stilbenoid, diarylheptanoid, and gingerol biosynthesis’, and DEGs of the CK vs. PC and PI vs. PP comparisons were significantly enriched in ‘ubiquinone and other terpenoid-quinone biosynthesis’. PAL plays important roles in secondary metabolism and defense response, and is often used as an important indicator of plant resistance [44]. Overexpression of *GmPAL2.1* can enhance the resistance of soybean against *Phytophthora sojae* [44]. Pepper *CaPAL1*, as a positive regulator of salicylic acid (SA)-dependent defense signals, inhibits microbial pathogens by enhancing PAL activity. Overexpression of *CaPAL1* increased the resistance of Arabidopsis to *Pseudomonas syringae* [45]. The Arabidopsis *pal1*/*pal2*/*pal3*/*pal4* quadruple knockout mutant is more susceptible to *P. syringae* [46]. Tobacco overexpressing PAL exhibited significantly reduced susceptibility to the fungal pathogen *Cercospora nicotianae* [47]. In this study, the 13 DEGs involved in the ‘L-phenylalanine catabolic process’ in the CK vs. PC comparison were all *PALs*. Of them, 12 *PALs* were highly expressed in the PC and PP groups, indicating that they actively participate in the defense response of gerbera against *P. cryptogea*, and *P. indica* can enhance the resistance of gerbera to root rot by modulating the transcription levels of *PAL* genes.

### 3.2. P. indica Enhances the Resistance of Gerbera to Root Rot by Up-Regulating Fatty Acid- and Oxylipin Biosynthesis-Related Genes

In this study, DEGs involved in the ‘oxylipin biosynthetic process’ were mostly significantly down-regulated in the PC vs. PP, CK vs. PI, and CK vs. PC comparisons, while they were up-regulated in the PI vs. PP comparison. Moreover, genes associated with the ‘linoleic acid metabolic’ pathway were mostly significantly down-regulated in the CK vs. PI, CK vs. PC, and PI vs. PP comparisons.

Research revealed that both mutualistic and pathogenic fungi could interfere with the fatty acid biosynthesis pathway to facilitate or promote their invasion into the host [48]. The transcription level of *fatty acid desaturase* (*FAD*) in *P. indica*-colonized tomato was significantly increased [5]. In banana, *P. indica* obviously alleviated the effects of *Foc*TR4 on several *MaFADs* and FA biosynthesis [49].

Oxylipins, pivotal catalytic products of the LOX-mediated reactions, play a vital role in plant growth and stress responses [50]. In this study, we found that both *P. indica* and *P. cryptogea* inoculation impacts the transcriptional levels of genes involved in the ‘alpha-linolenic acid metabolism’ and ‘linoleic acid metabolism’ pathways, especially *LOX* genes, which show a positive response to both fungi. LOX genes can activate plant defense against a variety of pathogens [51]. Overexpression of *CaLOX1* in pepper potentiates plant defense and cell death when responding to pathogen infection, while silencing *CaLOX1* mitigated the resistance of pepper to bacterial and fungal pathogens. In addition, overexpression of *CaLOX1* has been shown to confer *A. thaliana* with broad-spectrum resistance to a variety of pathogens [48]. In cotton, silencing *GhLOX2* greatly affected the JA signaling pathway and significantly suppressed the biosynthesis of lignin and accumulation of H_2_O_2_, resulting in increased susceptibility of cotton to *Verticillium dahliae* [52]. The expression of *MaLOXs* in banana is regulated by JA and *Foc*TR4, suggesting that *LOX* genes function in defense against *Fusarium* wilt pathogens via regulating the JA pathway [53]. Studies have also shown that overexpression of persimmon *DkLOX3* can promote biosynthesis of ethylene, which may confer resistance to necrotic pathogens by affecting the ET signaling pathway [54,55]. The massive reprogramming of fatty acid- and oxylipin metabolism-related genes in *P. indica* and *P. cryptogea* co-inoculated gerbera roots suggested that the JA and ET signaling pathways might be influenced massively by both of the two fungi.

### 3.3. P. indica Functions in the Gerbera–P. cryptogea Interaction by Influencing Hormone Signaling, MAPK Signaling, and TF Pathways

Plant response to pathogen infections is dependent on a complex network of interactions among different phytohormone signals. The function of phytohormones in plant–*P. indica* interactions has been well established. Its plant beneficial effects are mainly attributed to the biosynthesis and accumulation of phytohormones such as IAA, CTK, and GA, and enhanced plant resistance is closely related to systemic resistance involving stress phytohormones such as ABA, ET, SA, and JA [56]. *P. indica* promotes root elongation and lateral root development by increasing the content of IAA in Chinese cabbage roots [57]. In our previous study, the contents of IAA and GA in the roots of gerbera were found to be massively increased after *P. indica* colonization [16]. Our results also showed that the colonization of *P. indica* impacted the transcriptional abundance of hormone metabolic pathway-related genes and *TF* genes. Among the differentially expressed *TF* genes, many were also closely related to plant hormone signaling. For instance, the transcription levels of *PYL*, *ETR,* and *ERF* were up-regulated by *P. indica* and *P. cryptogea*, while the transcription levels of *ARF*, *IAA,* and *MYC* were down-regulated. ET and JA usually have a cooperative effect in plant defense responses. Our results suggest that *P. indica* may alter the expression of JA/ET-related genes by inducing transcriptional reprogramming of gerbera, thereby rapidly and effectively boosting the basic defense reaction against *P. cryptogea* infection [58]. JA is a hormone that uses α-linolenic acid as a biosynthesis precursor and itself as a signaling molecule that stimulates downstream stress responses [59].

Activating the MAPK cascade is one of the pivotal responses of hormone signal transduction and innate immunity [60]. This study found that the MAPK signaling pathway was significantly enriched by DEGs of CK vs. PC and PI vs. PP comparisons, indicating that the cascading pathway is a critical function in *P. indica*-enhanced disease resistance. ERF is involved in a variety of defense-related signaling pathways, such as MAPK cascades and plant hormone signal transduction. Overexpression of *ERF1* in Arabidopsis boosted resistance against necrotrophic fungi (*B. cinerea* and *Plectosphaerella cucumerina*) [61]. Among the 743 DE *TFs*, *AP2/ERFs* accounted for the predominant proportion, and an *ERF* gene (CL12783.Contig2_All) was a common DEG overlapped by the four comparisons. In response to *P. cryptogea* infection, the expression levels of many ET signaling-related genes, such as *ETR/ERS*, *MKK9*, *MPK3/6*, and *ERFs*, were significantly up-regulated. Apple (*Malus domestica*) MdERF110 can specifically bind the promoter of *MdPR4-2* and activate the gene’s expression, thereby ultimately enhancing the resistance of apple to *Alternaria alternata* [62]. Consistently, the expression of the ERF downstream disease resistance gene *PDF* was also up-regulated after the pathogen infection, and its encoded protein confers suppression of the growth of *P. cryptogea* and boosts the root rot resistance of gerbera [63]. Therefore, it was hypothesized that *P. indica* colonization could activate MAPK cascade signaling and boost the resistance of gerbera against root rot via regulating some JA/ET pathway-related genes. To confirm this hypothesis, we further analyzed the function of the *GhERF*. Due to the lack of transient and stable genetic transformation systems for gerbera, we employed a tobacco leaf-based transient overexpression method. The results demonstrated that the overexpression of *GhERF* obviously reduced lesions caused by *P. cryptogea* in tobacco leaves, and significantly up-regulated the expression of *NbPR2*, *NbACO,* and *NbLOX*, indicating that *GhERF* mainly inhibits the *P. cryptogea* infection through activating these defense-related genes.

## 4. Materials and Methods

### 4.1. Plant and Fungi Materials

In vitro-cultured ‘Linglong’ gerbera (*G. hybrida* cv. ‘Linglong’) seedlings that were cultured in rooting medium for 4 weeks were provided by the Institute of Flowers, Yunnan Academy of Agricultural Sciences. After being transplanted into nutrient soil (FAFARD^®^#1VC, Canada), gerbera seedlings were cultured in a greenhouse with a temperature of 25 ± 2 °C, a relative humidity of 60–80%, and a photoperiod of 12 h light/12 h dark (with light intensity of 3000 ± 200 lx on leaves) for six weeks before *P. indica* inoculation. The *Nicotiana benthamiana* plants used for leaf transient overexpression were cultured under the same culture conditions for 45 d. The *P. indica* strain (DSM11827) used in this study was kindly provided by Professor KaiWun-Yeh of Taiwan University and maintained in our laboratory. The *P. cryptogea* strain used in this study was isolated and preserved in our lab [16].

### 4.2. Fungal Inoculation Suspension Preparation

*P. indica* plugs were inoculated onto the center of a Petri dish with fresh potato dextrose agar (PDA) medium, and then cultured at 28 °C in the dark for 7 d. Two fresh *P. indica* plugs with a diameter of 5 mm were picked from colony edges, added to 300 mL of potato dextrose broth (PDB) medium, cultured via shaking at 200 rpm under 28 °C in the dark for 3 d, filtrated by six layers of gauze, and then adjusted to a concentration of 2 × 10^7^ spore/mL using PDB medium to get the *P. indica* inoculation suspension [15]. By using the same method, the *P. cryptogea* inoculation suspension was prepared and adjusted to a final concentration of 5 × 10^6^ spores/mL.

### 4.3. Fungal Inoculation

Healthy and uniform ‘Linglong’ gerbera plants were first classified into two groups: One group was watered with *P. indica* inoculation suspension every two days for three times (PI group), and the other group was treated the same way with an equal volume of PDB (CK group). Each group comprised a minimum of 50 gerbera plants. After fungal colonization detection using the Trypan Blue staining method, 15 gerbera plants from each of the CK and PI groups were inoculated with *P. cryptogea* inoculation suspension (PC and PP groups). When plants from the PC group exhibited typical root rot disease symptoms in leaves (about 12 days post *P. cryptogea* inoculation, dpi) [16], the roots of gerbera plants from the CK, PI, PC, and PP groups were harvested.

### 4.4. Transcriptome Sequencing

Total RNA was individually extracted from gerbera roots from the CK, PI, PC, and PP groups using the Trizol kit (Invitrogen, Carlsbad, CA, USA). After removing residual DNA with DNase I (RNase-free), the quality and concentration of RNA were detected by 1% agarose gel electrophoresis, a NanoPhotometer^®^ spectrophotometer (IMPLEN, Westlake Village, CA, USA), and the Bioanalyzer 2100 system (Agilent Technologies, Santa Clara, CA, USA). Next, the rRNA in the total RNA was removed using the Epicentre Ribo-zeroTM rRNA Removal Kit (Epicentre, Madison, WI, USA), and then used for cDNA library construction using the NEBNext^®^ UltraTM Directional RNA Library Prep Kit for Illumina^®^ (NEB, Ipswich, MA, USA). Each library was prepared using a mixture of root RNA from five plants. Twelve cDNA libraries (three replicates for each group) were sequenced on the BGISEQ-500 platform. After removing the low quality reads in the raw data, the remaining clean reads were used for transcriptome assembly by Beijing Liuhe Huada Gene Co, Ltd. (Beijing, China).

### 4.5. Identification and Enrichment Analysis of Differentially Expressed Genes (DEGs)

Gene expression level was computed as FPKM (fragments per kilobase of exon per million mapped fragments) for normalization. Differential expression analysis was performed by using DESeq2 with average FPKM ≥ 1, *q*-value < 0.05, and |log_2_ (fold change, FC)| ≥ 1 as criteria. Benjamini and Hotchberg’s False Discovery Rate method was applied for the adjustment of the *p*-values. Then, gene ontology (GO) and Kyoto Encyclopedia of Genes and Genomes (KEGG) enrichment analyses of DEGs from each comparison were performed using TBtools [64]. For the identification of significantly enriched GO terms and KEGG pathways, a corrected *q*-value < 0.05 was used as the criterion.

### 4.6. Gene Expression Validation Using Real-Time Quantitative PCR (RT-qPCR)

The same total RNA samples used for RNA-Seq were reversed-transcribed into cDNA using the PrimeScript^TM^ RT reagent Kit (Perfect Real Time) (Takara, Dalian, China). To verify the reliability of our RNA-Seq data, twelve DEGs were selected for further RT-qPCR validations (Table S12). Amplifications were performed on a LightCycler 480 real-time quantitative PCR instrument (Roche, Xiamen, China). By using *18S rRNA* as the reference to normalize gene expression [63], the relative expression levels of each selected gene in the roots of gerbera plants from the four groups were calculated using the 2^−ΔΔCT^ method [65].

### 4.7. Gene Cloning and Vector Construction

The RevertAid First Strand cDNA Synthesis (Thermo Scientific, Shanghai, China) kit was employed for reverse transcription. GhERF-specific primers were designed using DNAMAN version 9.0 software (forward and reverse primers contain *Xba*I and *Pst*I restriction site sequences, respectively). The PCR amplification program was as follows: pre-denaturation at 94 °C for 3 min; denaturation at 94 °C for 30 s; annealing at 56 °C for 30 s, extension at 72 °C for 60 s, 34 cycles, and then a final extension at 72 °C for 10 min. Then, PCR products were detected using 1.2% agarose gel electrophoresis; the gel was extracted using the Gel Extraction Kit (Omega, GA, USA), ligated into the pMD18-T vector, and then transformed into Escherichia coli DH5α component cells. PCR-positive clones were sent to the Fuzhou Qingke Biological Company (Fuzhou, China) for sequencing verification.

After digesting the pMD18-T-GhERF vector and pCAMBIA1301 using *Xba*I and *Pst*I, the gel extracted target *GhERF* CDS sequence was ligated into the pCAMBIA1301 vector to obtain the recombinant pCAMBIA1301-GhERF overexpression vector. Then, the pCAMBIA1301 empty vector and pCAMBIA1301-GhERF vectors were individually transformed into *Agrobacterium* GV3101 competent cells.

### 4.8. Tobacco Leaf Transient Transformation and P. cryptogea Resistance Assays

Agrobacteria carrying the pCAMBIA1301 empty vector/pCAMBIA1301-GhERF vector were cultured to OD_600_ = 1.5~2.0, and then centrifuged at 8000 rpm for 10 min to collect bacteria. Then, the agrobacteria were resuspended with inoculation buffer (10 mM/L MgCl_2_ + 10 mM/L MES + 100 μM/L acetosyringone (AS), pH = 5.8), adjusted to OD600 = 0.8–1.0, stored for 3 h at 28 °C in the dark, and injected into tobacco leaves according to Wang et al. [66]. Three biological replicates were performed for both the EV and pCAMBIA1301-GhERF vectors, and each biological replicate contained six leaves. After *Agrobacterium* inoculation, tobacco plants were placed in the dark in the greenhouse at 25 ± 2 °C with a relative humidity of 60~80% for 2 d. For pathogen inoculation, detached tobacco leaves were pricked with a needle and covered with *P. cryptogea* plugs with a diameter of 0.5 cm. After wrapping leaf petioles with moist cotton balls, inoculated tobacco leaves were placed in a Petri dish containing 3% agar and kept in a 25 ± 2 °C growth chamber with a photoperiod of 12 h light/12 h dark. Three days later, disease symptoms were observed and photographed. Then, P. *cryptogea*-inoculated tobacco leaves were divided into two groups. Leaves of one group were immersed in trypan blue dye solution (containing 10 mg of trypan blue powder + 10 g phenol + 20 mL ethanol + 10 mL lactic acid + 10 mL ultrapure water) in boiling water for 5 min, and then decolorized using chloral hydrate solution. After taking photos, the histogram tool in Image J was used to convert and calculate the lesion areas. Leaves of the other group were used for RNA isolation and RT-qPCR analysis of tobacco defense-related genes (*NbPR2*, *NbPR3*, *NbACO*, *NbLOX*, and *NbPDF1.2*) [67].

### 4.9. Statistics Analysis

All the results obtained in this study were calculated and analyzed using Excel 2010 and displayed as the average ± standard deviation (SD) of at least three replications. GraphPad Prism version 8.0 was used for significance analysis of the differences among the four groups using One-way ANOVA at the 5% level.

## 5. Conclusions

**Figure 8:**
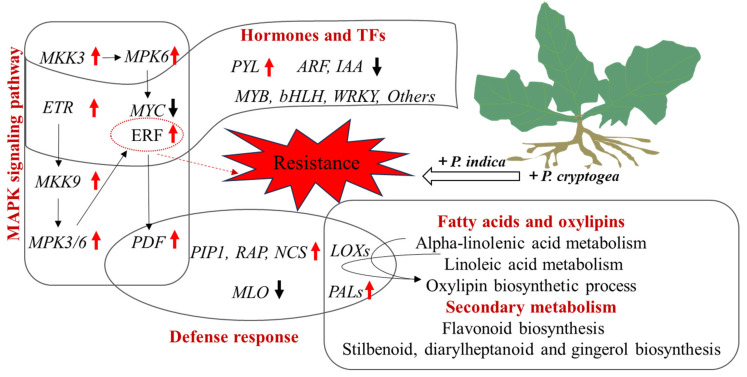
Schematic overview of the influences of *P. indica* and *P. cryptogea* on the root transcriptome profiling of gerbera. Red and black arrows represent up-regulated and down-regulated expression change patterns, respectively.

In this study, by employing comparative transcriptomics analysis, we revealed that *P. indica* colonization alleviated the pathogenicity of *P. cryptogea*, which was mediated by the up-regulation of genes associated with ‘linoleic acid metabolism’, ‘phenylpropanoid biosynthesis’, ‘hormone signal transduction’, and the ‘MAPK signaling pathway’ (Figure 8). Furthermore, the expression levels of genes encoding transcription factors and protein kinases were significantly influenced by *P. indica* and *P. cryptogea* (Figure 8), indicating that co-inoculation exerts a remarkable regulation on the gerbera transcriptome. Functional analysis of a common DEG, *GhERF*, showed that its transient overexpression significantly inhibited *P. cryptogea* infection and up-regulated the transcription of defense-related genes, demonstrating the role of *GhERF* in *P. indica*-enhanced disease resistance.

## Figures and Tables

**Table 1:** The 30 common DEGs identified in CK vs. PI, CK vs. PC, PI vs. PP, and PC vs. PP comparisons.

Gene Name	Gene ID	Arabidopsis ID	Mean FPKM of CK	Mean FPKM of PI	Mean FPKM of PC	Mean FPKM of PP
*SUD1*	CL11682.Contig1_All	AT4G34100 [23]	9.443	2.396	2.3	5.86
*ERF*	CL12783.Contig2_All	AT5G44210 [24,25]	22.443	7.91	7.696	30.27
*XAT3*	CL12943.Contig2_All	AT2G41640 [26]	24.906	5.63	6.28	25.633
*XAT3*	CL12943.Contig3_All		47.76	11.946	12.746	47.91
*Syntaxin 6*	CL14620.Contig2_All	AT2G18860 [27]	19.32	4.056	5.5	15.363
*APM1*	CL19393.Contig6_All	AT4G33090 [28]	9.99	3.406	2.25	6.166
*MGL*	CL19502.Contig11_All	AT1G64660 [29,30]	13.843	53.536	57.696	13.296
*LAC21*	CL19907.Contig5_All	AT5G21105 [31]	11.313	2.806	201.293	32.013
*LSAT*	CL2040.Contig1_All		2.196	8.563	14.483	2.606
*ROMT*	CL2802.Contig14_All		13.126	28.343	46.053	6.833
*ROMT*	CL2802.Contig4_All		5.073	15.94	33.096	3.593
*ROMT*	CL2802.Contig5_All		1.013	4.996	8.936	1.413
*Uncharacterized protein*	CL4667.Contig1_All		6.116	1.18	1.566	8.28
*Ccrd*	CL4667.Contig3_All		5.236	1.09	1.5	4.933
*F-box protein*	CL4972.Contig1_All	AT5G49610 [32]	106.416	28.013	21	69.43
*F-box protein*	CL4972.Contig2_All		182.543	46.41	38.53	120.03
*CPK16*	CL5372.Contig12_All	AT2G17890 [33]	58.646	27.49	20.296	49.31
*CPK18*	CL5372.Contig16_All	AT4G36070 [34]	42.99	20.686	16.793	44.936
*ZAT10*	CL730.Contig2_All	AT1G27730 [35,36,37]	40.54	18.61	19.503	63.74
*Uncharacterized protein*	CL8539.Contig2_All	AT5G04860 [38]	7.183	2.063	1.52	4.806
*Disease resistance protein*	CL905.Contig10_All	At5G66900 [39]	28.32	8.213	6.43	26.043
*Disease resistance protein*	CL905.Contig12_All		22.863	4.553	3.646	20.2
*Disease resistance protein*	CL905.Contig24_All		19.126	5.816	5.166	18.293
*Disease resistance protein*	CL905.Contig8_All		16.94	2.953	1.95	14.54
*CYP73A1*	Unigene118068_All	AT4G35300 [40]	1.396	10.636	5.503	1.436
*POD1*	Unigene21498_All	AT1G67960	1.816	7.993	5.493	1.16
*Acyltransferase-like protein*	Unigene44933_All	AT1G54570 [38]	11.226	2.283	2.546	15.003
*FAO2*	Unigene45366_All	AT4G28570 [23]	5.68	1.8	11.61	21.68
*LOX3.1*	Unigene45770_All	AT1G17420 [41]	93.853	13.326	40.846	104.526
*XRQ*	Unigene61097_All		5.9	1.68	41.55	4.396

## Data Availability

Data will be made available upon request. The BioProject accession number of our raw transcriptome data is PRJNA1471993, which can be checked at the following website link: https://www.ncbi.nlm.nih.gov/sra/PRJNA1471993 (accessed on 6 May 2026).

## Supplementary Materials

The following supporting information can be downloaded at: https://www.mdpi.com/article/10.3390/plants15121771/s1, Figure S1: Enrichment analysis results of up- and down-regulated DEGs. A: GO enrichment analysis of up- and down-regulated DEGs; B: KEGG enrichment analysis of up- and down-regulated DEGs; Figure S2: Expression heat maps of LOXs related to the ‘linoleic acid metabolism’ pathway in the four comparison groups; Table S1: Statistics analysis results of transcriptome sequencing data; Table S2: DEGs identified in the CK vs. PI comparison; Table S3: DEGs identified in the CK vs. PC comparison; Table S4: DEGs identified in the PI vs. PP comparison; Table S5: DEGs identified in the PC vs. PP comparison; Table S6: GO enrichment analysis of DEGs in four comparisons; Table S7: GO enrichment analysis of the up-regulated DEGs in four comparisons; Table S8: GO enrichment analysis of the down-regulated DEGs in four comparisons; Table S9: KEGG enrichment analysis of DEGs in four comparisons; Table S10: KEGG enrichment analysis of the up-regulated DEGs in four comparisons; Table S11: KEGG enrichment analysis of the down-regulated DEGs in four comparisons; Table S12: Information for the primers used in this study.

## Abbreviations

The following abbreviations are used in this manuscript:

ABA	Abscisic acid
ALDH2C4	Aldehyde dehydrogenase family 2 member C4
ACO	1-aminocyclopropane-1-carboxylic acid oxidase
AMF	Arbuscular mycorrhizal fungus
ARF	Auxin response factor
BP	Biological process
CC	Cellular component
CK	Non-inoculated control
CPI	Cysteine protease inhibitor
DJ1D	DJ-1 protein homolog D
dpi	Days post inoculation
DEG	Differentially expressed gene
ERF	Ethylene response factor
ET	Ethylene
FAD	Fatty acid desaturase
FC	Fold change
FPKM	Fragments per kilobase of exon per million mapped fragments
GA	Gibberellic acid
GLU	β-Glucosidase
GO	Gene ontology
IAA	3-indoleacetic acid
JA	Jasmonic acid
KEGG	Kyoto Encyclopedia of Genes and Genomes
LBD	Lateral organ boundaries domain protein
LOX	Lipoxygenase
MAPK	Mitogen-activated protein kinase
MF	Molecular function
MLO	Mildew resistance locus O
NCS	Norcoclaurine synthase
PAL	Phenylalanine ammonia lyase
PC	*P. cryptogea*-inoculated
PDA	Potato dextrose agar
PDB	Potato dextrose broth
PDF	Plant defensin
PI	*Piriformospora indica*-colonized
PIPs	PAMP-induced secreted peptides
PR	Pathogenesis-related protein
PSK	Phytosulfokine
RAN2	RAS-related GTP-binding nuclear protein 2
RT-qPCR	Real-time quantitative PCR
RAP	Root allergen protein
TF	Transcription factor
TBL	Trichome birefringence-like
