# Supplementary figures and images for: Comparative Transcriptomic Analysis Reveals the Underlying Mechanism of Piriformospora indica-Enhanced Root Rot Resistance in Gerbera hybrida
Source: Plants (Basel). 2026 Jun 8;15(12):1771. doi: 10.3390/plants15121771 (PMC13306804; doi:10.3390/plants15121771)

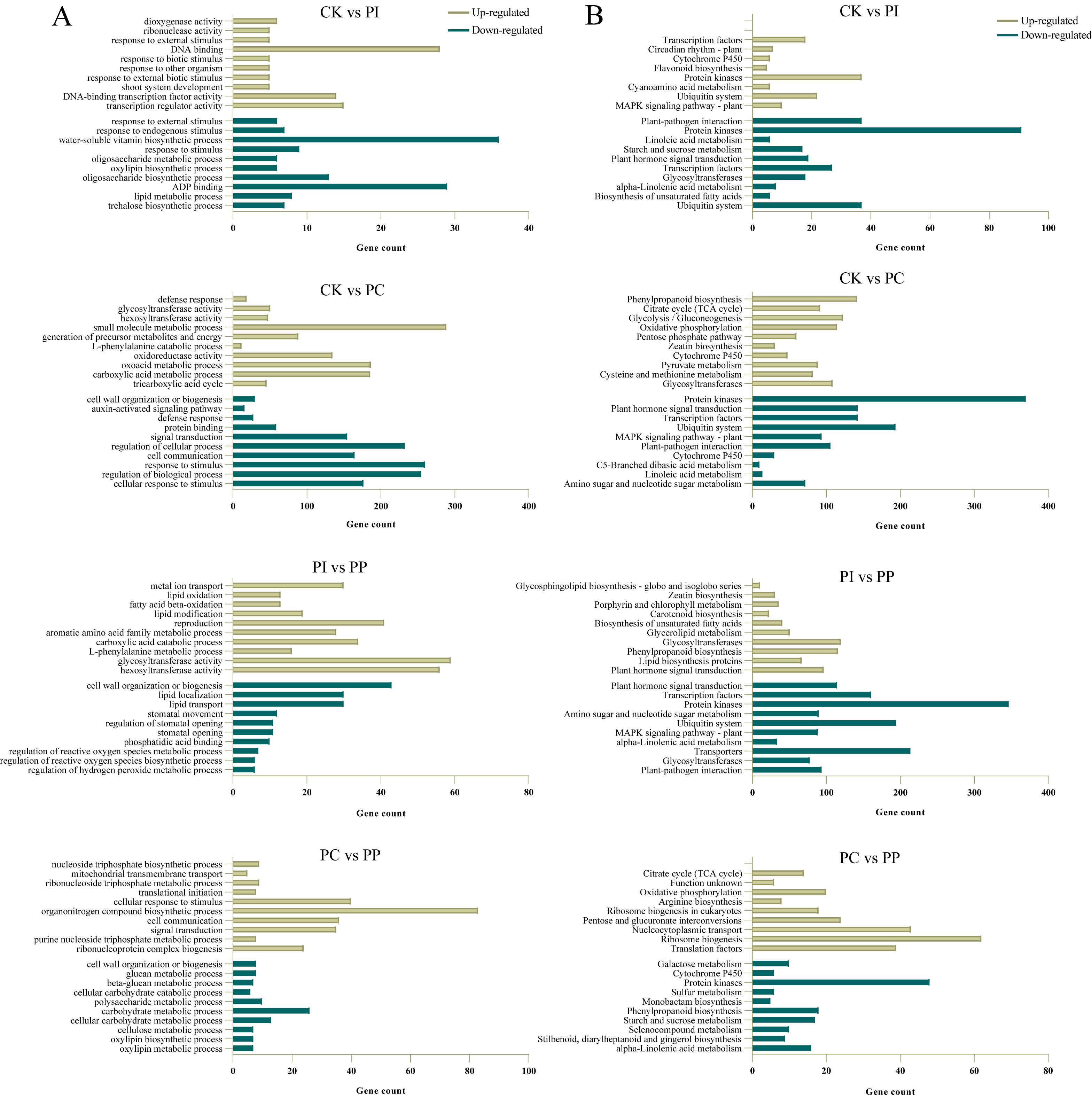

Supplement: Supplementary file 1 [file plants-15-01771-s001.zip › Figure S1.png]

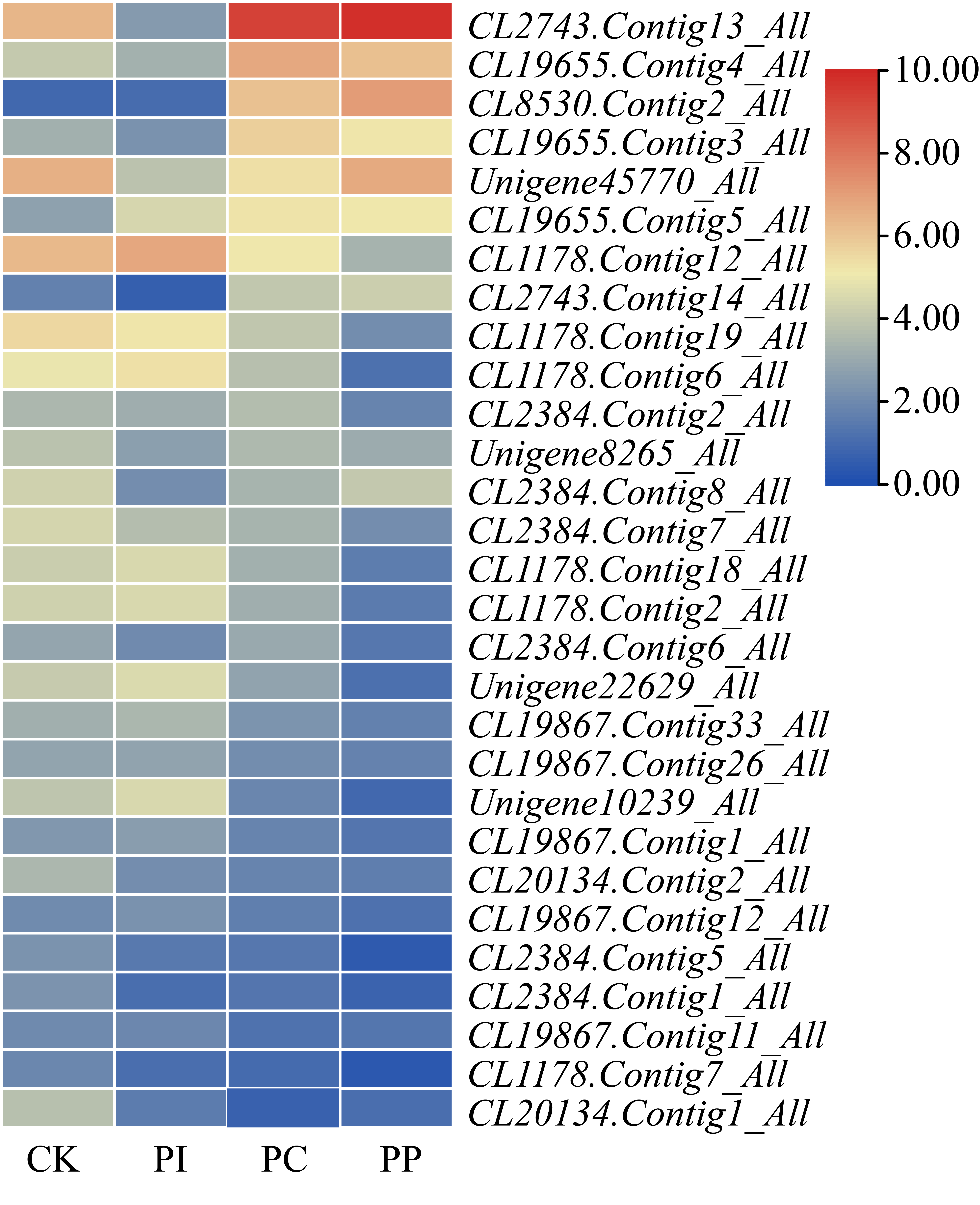

Supplement: Supplementary file 1 [file plants-15-01771-s001.zip › Figure S2.png]
